# Subthreshold yellow laser for fovea-involving diabetic macular edema in a series of patients with good vision: effectiveness and safety of a fovea-sparing technique

**DOI:** 10.1186/s12886-020-01536-4

**Published:** 2020-07-06

**Authors:** Alejandro Filloy, Victor Chong, Eduard Solé

**Affiliations:** 1 Ophthalmology department, Joan XXIII University Hospital, Rovira i Virgili University, Mallafré i Guasch, 4, 43002 Tarragona, Spain; 2 Arnott Eye Associates, London and University of Oxford, Oxford, UK

**Keywords:** Autofluorescence, Diabetic, Fovea, Subthreshold, Yellow

## Abstract

**Purpose:**

Patients with center-involved diabetic macular edema (CI-DME) with good visual acuity (VA) represent a controversial clinical scenario in which a subthreshold laser might be a reasonable approach. We report a case series of patients with CI-DME with VA better than 20/32 who were treated with a subthreshold 577 nm (yellow) laser.

**Methods:**

The area of retinal thickening on OCT was treated with confluent laser spots at individually titrated power. The fovea was spared from treatment. Effectiveness and safety were evaluated through OCT and autofluorescence (AF) as well as BCVA.

**Results:**

A total of 23 eyes from 19 patients were treated. VA ranged from 20/20 to 20/30. The follow-up period ranged from 6 to 18 months. Edema in OCT resolved completely at the end of follow-up in 56.5% (13/23) of the cases. Central retinal thickness was reduced at 12 weeks and at the end of follow-up, with a mean reduction of 16.9 μm and 22 μm, respectively (paired t-test *p* = 0.001 and 0.0003). VA remained stable. The laser was invisible (OCT, AF, Fundoscopy) in 91,3% (21/23) of eyes.

**Conclusions:**

A fovea-sparing yellow subthreshold laser was safe and effective for treating CI-DME patients with good VA in this case series. This technique is of interest to prevent the progression of mild edema and might avoid or reduce the use of more invasive and expensive therapies. Excluding the fovea from the treated area does not seem to affect the results, which is of interest to novel laser practitioners.

## Background

Diabetic macular edema (DME) is a complex disease that presents itself in a wide variety of clinical scenarios that drive the need for individualized treatment [1]. One controversial setting involves patients who retain good visual acuity (VA) but display early center-involved DME (CI-DME) on OCT. Recently, DRCR.net has assessed whether anti-VEGF therapies would be useful in this situation versus observation and laser photocoagulation [2]. However, anti-VEGF is still invasive and expensive, so at least to the present day, close observation has become the standard of care, as the retinal damage caused by conventional lasers is becoming increasingly unacceptable to patients and retinal specialists [3, 4]. The use of a subthreshold laser (STL), which does not cause cicatricial or tissue-changing effects, is considered a safe technique to work around and even on the fovea [5–9] since no clinically visible effects appear on the fundus, optical coherence tomography (OCT) or autofluorescence (AF), the latter being especially sensitive to any disturbances/damages in the retinal pigment epithelium (RPE) [10]. The latest developments in STL, such as the partly or fully automated delivery systems that guarantee that the edematous area is treated densely enough, help increase its efficacy, thus making this technique more attractive [11, 12]. We present a study on the effectiveness of a subthreshold 577 nm (yellow) subliminal laser on VA and OCT and safety on AF for the treatment of CI-DME patients with good VA (20/32 or better). In this study, we administered the laser through a “fovea-sparing” technique, which avoids delivering laser treatment on the fovea itself and up to 100 μm from the fovea.

## Methods

This was a retrospective consecutive case series. The inclusion criterion was OCT-confirmed CI-DME. To qualify for CI-DME, OCT had to display intraretinal cysts (IRC) with or without intraretinal exudates (IRE) or neurosensory detachment (NSD) in the central 1 mm subfield. The patients were asymptomatic (without any VA deterioration, metamorphopsia or other newly acquired visual symptoms) with a best corrected visual acuity (BCVA) better than 20/32. Patients who had received treatment for DME in the three months prior to baseline were excluded.

Swept-Source OCT (Triton, Topcon, Japan) was employed. AF was taken using the same device and fundus photography was also taken at baseline and at every follow-up visit. Treatment was delivered through a yellow wavelength (577 nm) subthreshold laser (Subliminal Easyret, Quantel Medical, Cournon d’Auvergne, France). Treatments were performed by a single surgeon (A. F). The standard parameters were 5% duty cycle, spot size 160 μm in diameter and 0.2 s in duration. The power to employ was titrated case by case at 1/3 of the minimum power needed to produce a barely visible whitening of the healthy retina in the macular periphery. Laser spots were delivered in a confluent manner to cover the entire edematous area on the OCT thickness map as well as 200 μm of flat retina around this area. The fovea itself and 100 μm of the surrounding area were spared from treatment. Before discharge, the patients were instructed to consult us if they noticed any symptoms. They were scheduled for evaluation with VA, OCT and AF at 12 weeks after STL and every 12 weeks thereafter.

The collected data were analyzed with IBM SPSS statistics 25.0 (IBM, USA). Statistical significance was considered with *p* values of at least 0.05.

## Results

**Fig. 1:**
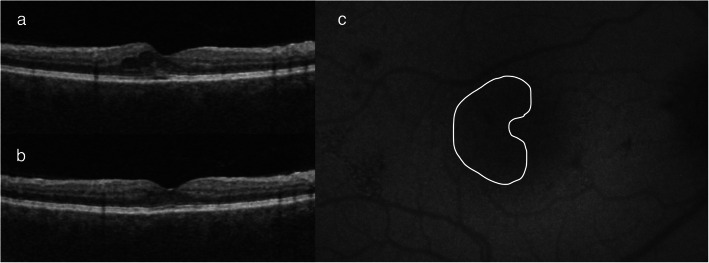
This asymptomatic patient was diagnosed with DME during routine examination. Intraretinal cysts and early neurosensory detachment are visible. The CRT at this point was 302 μm. **a**. Twelve weeks after STL treatment, there was a marked improvement, with a decrease in CRT to 283 μm (**b**), as well as a lack of visible laser reaction on AF (**c**). Vision remained 20/20 at all times. The demarcated area on AF shows the treatment zone (also in Fig. 2). The OCT protocol for these images was a macular cube 9 × 9 mm

**Fig. 2:**
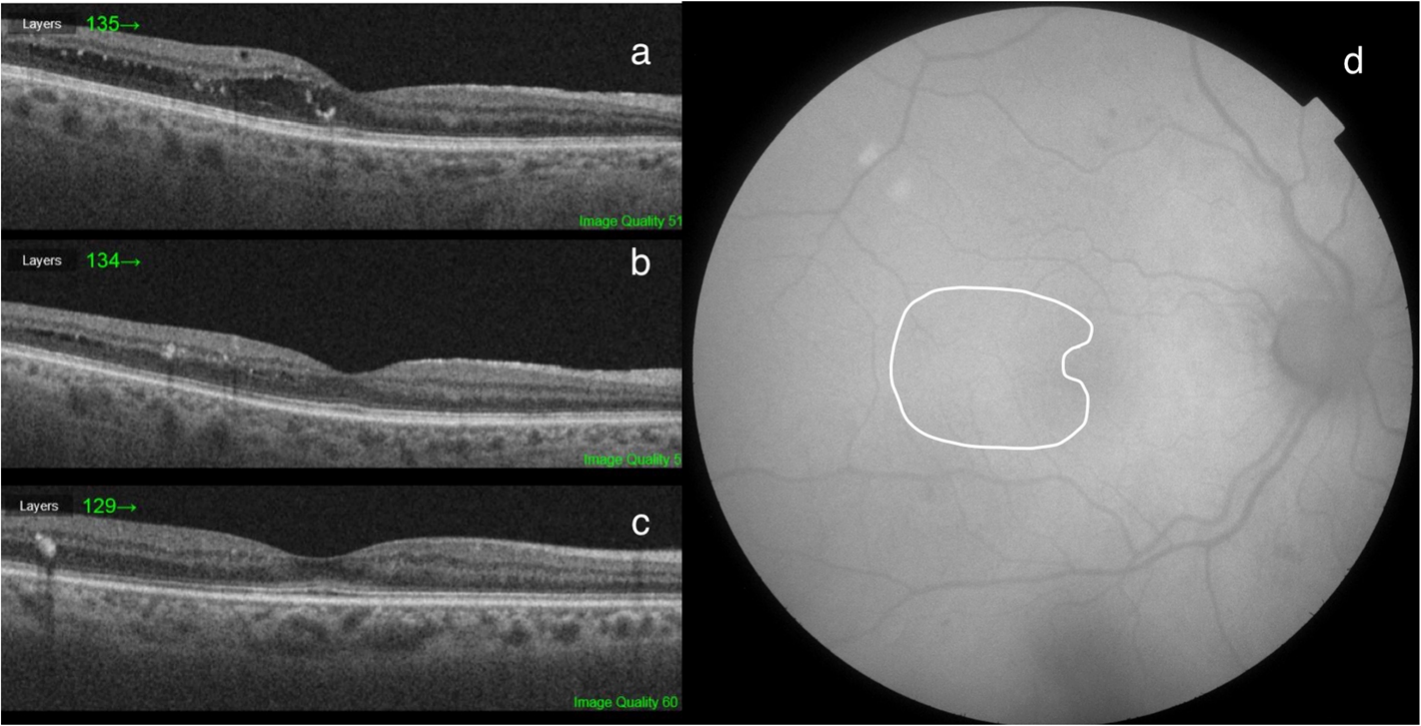
This patient showed remarkable improvement at the first post-laser visit (**b**, 12 weeks). At the next follow-up visit, we observed a slight additional improvement (**c**, 24 weeks). AF (**d**) does not show RPE disturbance. VA remained 20/25. CRT decreased from 282 μm at baseline to 266 μm at the first follow-up visit and finally 252 μm

A total of 23 eyes (19 patients) met the inclusion criteria (Table 1). The mean age was 61,3 years, and 15 patients had type-II diabetes mellitus (DM) while 4 had type I DM. Six of the patients were treatment naive, while thirteen received previous treatment for DME (7 received anti-VEGF, 5 focal laser, 1 both). The initial VA ranged from 20/20 to 20/32, averaging 20/25. The follow-up period ranged from 6 to 18 months (average 12.5 months). The mean power used was 396 mW, and the mean number of spots delivered was 288. VA after treatment remained stable (*p* = 0.16, paired t test), with no patients showing VA loss. Edema in OCT resolved completely in 30.4% (7/23) of the cases, while another 52.2% (12/23) showed improvement at the first post-laser visit. The total number of resolved cases increased to 56.5% (13/23) at the end of the follow-up. The OCT-measured central retinal thickness (CRT) decreased from 301 to 284 μm on average, a total of 17 μm, after the first 12 weeks (*p* = 0.001, paired t test). At the end of the follow-up, the average CRT decrease was 22 μm (*p* = 0.0003, paired t test) (Figs. 1 and 2). One case showed a further increase in CRT (+ 38 μm) without VA loss. None of the patients reported earlier than the scheduled visit for VA loss or the presence of any significant symptoms. Laser treatment was invisible on Fundoscopy, OCT and FA in 91.3% (21/23) of the cases. The 2 cases where the highest power was used displayed some visible spots as hyperautofluorescent discrete dots on AF as well as mild RPE disturbance on OCT; these 2 patients did not report any symptoms even on direct questioning. No other secondary effects were noted.

**Table 1:** Results

Patient	Eye	DM	OCT	PreL CRT	PostL CRT (12 week)	Last PostL CRT	Decrease in CRT	Power (mW)	Spots	Follow up (months)	Changes on AF	PreL VA	PostL VA
1	RE	II	IRC	288	257	250	38	450	350	18	NO	20/22	20/22
1	LE	II	IRC	280	265	263	17	450	460	10	NO	20/22	20/22
2	LE	I	IRC	296	284	277	19	325	80	15	NO	20/22	20/20
3	RE	I	IRC, NSD	302	283	276	26	350	150	9	NO	20/20	20/20
4	RE	II	IRC	303	268	247	56	350	200	10	NO	20/22	20/22
5	RE	I	IRC, IRE	301	288	295	6	400	60	12	NO	20/25	20/22
5	LE	I	IRC	287	272	251	36	300	180	9	NO	20/32	20/32
6	RE	II	IRC	269	274	254	15	350	200	14	NO	20/28	20/28
7	LE	II	IRC, IRE	275	267	270	5	500	180	15	YES	20/28	20/25
8	RE	II	IRC	258	226	240	18	400	200	15	NO	20/28	20/25
8	LE	II	IRC, IRE	243	230	230	13	500	180	17	YES	20/32	20/25
9	LE	II	IRC, IRE	270	260	260	10	450	250	13	NO	20/32	20/25
10	RE	I	IRC	376	350	323	53	300	480	9	NO	20/20	20/20
10	LE	I	IRC, NSD	510	431	442	68	300	560	9	NO	20/20	20/20
11	RE	II	IRC, IRE	355	393	393	−38	400	380	6	NO	20/32	20/25
12	RE	II	IRC	335	333	332	3	382	350	9	NO	20/28	20/28
13	LE	II	IRC, IRE	254	226	226	28	325	300	7	NO	20/22	20/22
14	RE	II	IRC, IRE	324	320	322	2	300	400	7	NO	20/22	20/22
15	LE	II	IRC	341	295	293	48	425	160	12	NO	20/32	20/25
16	LE	II	NSD	288	256	234	54	450	450	12	NO	20/25	20/20
17	LE	II	IRC, IRE	282	266	252	30	350	400	9	NO	20/20	20/20
18	RE	II	IRC, IRE	259	250	242	17	350	300	9	NO	20/20	20/20
19	LE	II	IRE	226	226	226	0	700	350	9	NO	20/28	20/28

*DM* Diabetis Mellitus, *PreL* Pre Laser, *PostL* Post Laser, *CRT* Central retinal thickness, *AF* Autofluorescence, *VA* Visual acuity, *IRC* Intraretinal cysts, *NSD* Neurosensory detachment, *IRE* Intraretinal exudates

## Discussion

The use of a subthreshold laser for the treatment of DME patients is known to lead to better results in terms of effectiveness and safety than conventional laser photocoagulation [13]. It might also decrease the injection burden in these patients [14]. In our study, a yellow subthreshold laser applied with a fovea-sparing technique with 1/3 power titration was shown to be a safe and effective treatment for CI-DME in patients presenting with good VA. In addition to preserving their VA, 95.7% (22/23) of the treated eyes improved to either complete or partial anatomical normalization at the last follow-up, which is likely to prevent VA loss in the near future.

CI-DME with good VA is a controversial clinical setting because of the disadvantages of the most commonly applied treatment strategies [3]: observation, which may lead to further deterioration with VA loss, and intravitreal therapy, which has a high cost and potential complications such as endophthalmitis that, although rare [15], is especially devastating when treating asymptomatic patients.

Recently, the DRCR network assessed this controversy [2] in a two-year randomized study comparing three arms: aflibercept, laser photocoagulation and observation, the last two receiving aflibercept as needed in the case of visual deterioration. The study concluded that observation is a reasonable option considering that there was a lack of significant differences in visual acuity between the three arms at the end of the study. It must be noted, however, that up to 34% of the patients in the observation group and 25% in the laser photocoagulation group required treatment with aflibercept at some point during the study (a median of 7 injections per patient over 2 years). None of our patients needed intravitreal therapy due to a deterioration in VA so far. In addition, the laser technology employed in that study was traditional ETDRS laser photocoagulation instead of subthreshold technology. It must also be noted, however, how the number of patients requiring aflibercept was lower in the laser arm than in the observation arm.

The safety of a transfoveal 810 nm (infrared) subthreshold laser has been previously evaluated in a similar clinical setting [9]. The results we obtained with the fovea-sparing technique and 577 nm wavelength were effective, and AF further supported the safety of this approach. Previous studies failed to demonstrate differences in outcomes between infrared and yellow wavelengths for the treatment of mild DME [16]. Treating the fovea directly remains controversial and is considered a risk for these asymptomatic patients with good vision. Success on the subthreshold relies on treating a large surface area, thus stimulating enough RPE cells to produce a clinically significant response [17, 18]. Since the fovea and its surroundings represent a small surface, excluding it from treatment means 3 or 4 fewer 160 μm spots at most, which will hardly be relevant when the average numbers of these treatments are usually around the hundreds. The therapeutic effect of treating the neighboring area will likely extend to the fovea. We believe that the fovea-sparing technique is of particular interest, as STL is gaining interest among retinal specialists. This makes the transition from conventional laser to STL easier, as novel STL surgeons can become familiar with the new parameters and delivery systems without the fear of the potentially devastating effect of a foveal or near-foveal laser burn.

We prefer individual titration to the use of standardized power to all patients to try to provide a treatment intense enough while remaining at the subthreshold. The reason we used 160 μm spots was to reach a compromise between the more widely used 200 μm (avoids relatively large unstimulated areas for missed spots) and 100 μm (takes much longer to treat the same area with smaller spots) spots. We employed the 5% duty cycle, which is currently the standard [6].

The two cases with visible spots on AF were probably related to an overestimation of power during titration. The patient with an increase in retinal thickness despite treatment presented 3 large microaneurysms within the thickened retina. The exudation coming from these relatively large lesions might require more than one session of STL.

## Conclusions

STL has been shown to be a safe and effective treatment alternative in this case series of CI-DME patients. The traditional observation and intravitreal treatment options present their own limitations. Excluding the fovea from treatment does not seem to impair the results compared to those in previous studies in similar patients. The main limitations of the present study are its retrospective nature, the relatively small number of patients included and the relatively short follow-up. A randomized prospective study to compare observation, STL and intravitreal anti-VEGF should be considered to firmly establish the usefulness of this technique to provide a more affordable and safer treatment for these asymptomatic patients.

## Abbreviations

AF: Autofluorescence
BCVA: Best Corrected Visual Acuity
CI-DME: Center-Involving Diabetic Macular Edema
CRT: Central Retinal Thickness
DME: Diabetic Macular Edema
IRC: Intraretinal Cysts
IRE: Intraretinal Exudates
NDS: Neusosensory Detachment
OCT: Optical Coherence Tomography
RPE: Retinal Pigment Epithelium
STL: Subthreshold Laser
VA: Visual Acuity
VEGF: Vascular Endothelial Growth Factor
